# General Anaesthesia Shifts the Murine Circadian Clock in a Time-Dependant Fashion

**DOI:** 10.3390/clockssleep3010006

**Published:** 2021-01-26

**Authors:** Nicola M. Ludin, Alma Orts-Sebastian, James F. Cheeseman, Janelle Chong, Alan F. Merry, David Cumin, Shin Yamazaki, Matthew D. M. Pawley, Guy R. Warman

**Affiliations:** 1 Department of Anaesthesiology, School of Medicine, University of Auckland, 1142 Auckland, New Zealand; n.ludin@auckland.ac.nz (N.M.L.); a.orts-sebastian@auckland.ac.nz (A.O.-S.); j.cheeseman@auckland.ac.nz (J.F.C.); j.chong@auckland.ac.nz (J.C.); a.merry@auckland.ac.nz (A.F.M.); d.cumin@auckland.ac.nz (D.C.); M.Pawley@massey.ac.nz (M.D.M.P.); 2 Department of Neuroscience, Peter O’Donnell Jr. Brain Institute, University of Texas Southwestern Medical Center, Dallas, TX 75390, USA; Shin.Yamazaki@UTSouthwestern.edu; 3 School of Natural and Computational Sciences, Massey University, 0745 Auckland, New Zealand

**Keywords:** general anaesthesia, circadian clock, phase response curve, PERIOD2::LUC, clock gene

## Abstract

Following general anaesthesia (GA), patients frequently experience sleep disruption and fatigue, which has been hypothesized to result at least in part by GA affecting the circadian clock. Here, we provide the first comprehensive time-dependent analysis of the effects of the commonly administered inhalational anaesthetic, isoflurane, on the murine circadian clock, by analysing its effects on (a) behavioural locomotor rhythms and (b) PER2::LUC expression in the suprachiasmatic nuclei (SCN) of the mouse brain. Behavioural phase shifts elicited by exposure of mice (n = 80) to six hours of GA (2% isoflurane) were determined by recording wheel-running rhythms in constant conditions (DD). Phase shifts in PER2::LUC expression were determined by recording bioluminescence in organotypic SCN slices (n = 38) prior to and following GA exposure (2% isoflurane). Full phase response curves for the effects of GA on behaviour and PER2::LUC rhythms were constructed, which show that the effects of GA are highly time-dependent. Shifts in SCN PER2 expression were much larger than those of behaviour (c. 0.7 h behaviour vs. 7.5 h PER2::LUC). We discuss the implications of this work for understanding how GA affects the clock, and how it may inform the development of chronotherapeutic strategies to reduce GA-induced phase-shifting in patients.

## 1. Introduction

The circadian clock is fundamental to human well-being as it regulates almost all aspects of our daily biochemistry, physiology, and behaviour including sleep, immune function, wound healing, and responses to drugs [1,2]. Disruptions of the circadian clock have been shown to have major impacts on health and well-being including metabolic disorders and diabetes, cancer, heart disease, and impaired immune function [1].

In mammals, the central circadian clock is located in the suprachiasmatic nuclei (SCN) of the hypothalamus [3]. Peripheral clocks that regulate local rhythms in physiology and biochemistry exist in almost all tissues of the body; however, these clocks remain under the coordination of the central SCN-based pacemaker [4].

The fundamental biochemical basis of circadian rhythm generation relies on a transcription-translation feedback loop of “clock genes”. In mammals, these genes include the positive regulatory elements *Clock*, and *Bmal1*, and the negative elements *Period 1* (*Per1*), *Period 2* (*Per2*), *Period 3* (*Per3*), and *Cryptochrome 1* (*Cry1*) and *2* (*Cry2)*. The protein products of these genes interact to generate subcellular oscillations of PERIOD and CYPTOCHROME proteins, which directly drive output rhythms in biochemistry, physiology, and behaviour [5].

These endogenous rhythms are “entrained” on a daily basis by geophysical cycles (primarily light) such that the period of the entrained rhythm is 24 h, and the phase relationship of the clock to that of the daily light cycle times events to occur at an appropriate time of the day. While light is the primary zeitgeber that shifts circadian clocks, numerous other agents, including drugs, can also exert a shifting effect on our circadian systems.

Following general anaesthesia, patients frequently experience sleep disruption and fatigue, which has been hypothesized to result at least in part, by general anaesthesia affecting the circadian clock [6]. There is increasing interest in the effects general anaesthesia may have on the circadian clock, and how this may impact on patients’ post-operative recovery [7]. While the way in which general anaesthetics work to exert their effects on consciousness is very poorly understood, it is clear that general anaesthesia (GA) has strong effects on the main neurotransmitter systems involved in the control of the circadian network and its entrainment to the light–dark cycle (e.g., Gamma aminobutyric acid/N-methyl-D-aspartate (GABA/NMDA)) [8]. Thus, it would seem plausible that GA could exert its action on the clock by interfering with the correct functioning of the circadian system.

Most SCN neurons are GABAergic and express GABA_A_ receptors [9]. The activation of GABA_A_ (using the agonist muscimol) can cause suppression of *Per1* and *Per2* expression in the SCN during the subjective day (CT6) [10], and light and GABA have been shown to have opposing effects on *Per1* expression in the subjective night [11]. Furthermore, the inhalational anaesthetic sevoflurane (which exerts it effect at least in part through GABA) has been shown to reduce the acetylation of the E-box regulatory region of the clock gene *Per2*, and to subsequently reduce CLOCK protein binding to the *Per2* E-box [12]; a potential mechanism via which GA may phase shift the clock. In addition, the fact that GA can affect sleep centres such as the ventrolateral preoptic nucleus (VLPO) and autonomic activity may also mediate its effect on rhythms.

Investigation of the effects of anaesthesia-induced clock disruption in the clinical setting is confounded by patients’ surgery and clinical management, and the fact that their underlying disease state disrupts circadian rhythms and sleep at baseline. Thus, animal models currently afford the best approach to studying anaesthesia-induced clock shifting.

Currently, there are in excess of 25 published studies demonstrating that anaesthetics can disrupt the circadian clock and alter circadian rhythms [7]. These include three papers in 2020, which demonstrate an effect of inhalational anaesthetics on behaviour and clock gene expression in the mouse [13], and possible effects of melatonin [14] and 5-HT7 serotonin receptors [15] on inhalational anaesthesia-induced circadian disruption in mice. Results from these studies are, however, largely contradictory, and effects at the molecular and behavioural levels often cannot be reconciled. For example, in a recent paper by Imai et al., [13] phase delays in behaviour (at ZT 12) are accompanied by increased expression of all of the elements (both positive and negative) of the molecular feedback loop. The use of varying protocols in which anaesthesia is only administered at isolated, and somewhat arbitrary times, and the lack of rigorous experimental controls [7] makes it difficult to consolidate the effects of GA on the clock, and to assess their clinical significance.

Previous studies from our group on the effects of GA on circadian rhythms in diurnal invertebrate models (the honey bee *Apis mellifera* and the fruit fly *Drosophila melanogaster*) clearly indicate that anaesthesia with the inhalational agent isoflurane causes robust shifts of behavioural and clock gene expression rhythms in a highly time–dependent manner [6,16]. Profound shifts occur in response to anaesthesia at concentrations of 1.5% and above, and durations three hours or longer [6,16].

While in bees, isoflurane-induced phase delays occur in both gene expression and behaviour during the subjective morning [6,17], in flies, daytime GA induces phase advances, and phase delays result from night-time GA (a time at which GA induced no shift in the honey bee) [16].

Here, we provide the first comprehensive time-dependent analysis of the effects of the commonly administered inhalational anaesthetic, isoflurane, on the murine circadian clock, by analysing the effects of this agent on behaviour and on PER2::LUC expression in the suprachiasmatic nuclei of the mouse brain. Taking advantage of a powerful transgenic *Per2* clock gene reporter system, and using standard anaesthetic protocols and animals of the same genetic background, these experiments, conducted at all times of the day and night, have enabled us to calculate full “phase response curves” for the effects of GA on behaviour and clock gene expression in mice, and to draw conclusions about how anaesthesia may be affecting the circadian system in this central mammalian model.

## 2. Results

### 2.1. Effects of GA on Locomotor Rhythms of C57 Mice

Of the 84 C57 mice used for the behavioural experiments, all animals survived to receive their initial intervention (either a 10 min “control” or six hour “treatment” GA with isoflurane). Three of the 84 animals died following the six-hour general anaesthetic intervention, and one further animal did not display rhythmic behavioural activity prior to its six-hour anaesthetic (and, thus, did not provide analysable data). A further two animals in the group that received the “control” (10 min) anaesthetic as a second intervention did not show rhythmic behavioural data following this anaesthetic intervention. Thus, 80 animals provided usable data for the “treatment” (six hour) GA, and 78 animals provided usable data for the “control” (10 min) GA.

**Figure 1:**
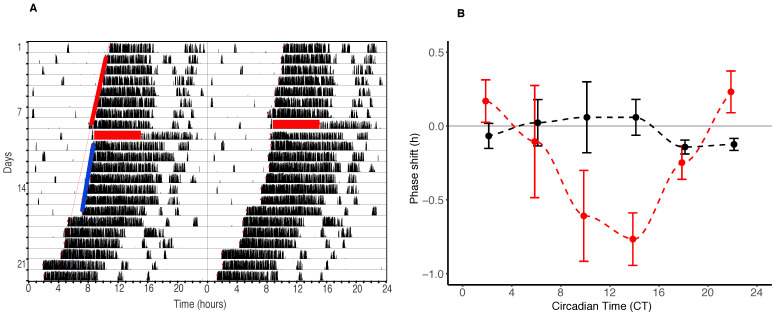
(**A**) Double plotted actogram of a single C57 BL6/VJU mouse (Mouse 70) recorded in constant conditions (DD) for a period of eight days prior to a single six-hour GA with isoflurane (2% in air) (red box) showing a clear phase delay in behavioural rhythms following anaesthesia. (**B**) Phase response curves for the effects of six hours of isoflurane (red line) (n = 80) and 10 min of isoflurane (control) (black line) (n = 78) on the clock at different Circadian Times (in four hour bins). Circadian Time of GA is defined as the time from onset of activity (CT12) until the midpoint of the six-hour GA. Data points are bin means (SEM). Control values have been slightly shifted (horizontally) for visualization. Control values have been horizontally moved for visualization. Dashed lines represent the cubic spline between means.

Persistent and reproducible shifts in the phase of locomotor activity rhythms were observed in response to isoflurane anaesthesia administered in constant darkness (Figure 1A,B, Table 1, Supplementary Table S1, Supplementary Figure S1), with no effect on the underlying period of the clock (average free-running periods changing less than 0.025 h following GA (Mean pre-GA τ = 23.72 h (±0.02 h) and post-GA = 23.75 h (±0.02 h).

Behavioural phase shifts following a six-hour treatment with isoflurane show a typical “weak” (Type One) pattern, with a pronounced delay portion centred around activity onset (CT12) (Figure 1B). Maximum phase advances of 0.23 h (±0.14 h) occurred between CT22–CT2 and a much longer and more pronounced delay portion between CT 6 and 18 showed a maximum delay of −0.77 h (±0.18 h) (Figure 1B). The largest individual phase shift was displayed by an animal anaesthetized at CT15, whose behavioural rhythms were delayed by −1.69 h.

Phase shifts in response to the “control” (10 min) anaesthesia were minimal (with maximum advances and delays of 0.06 h (±0.24 h) and −0.14 h (±0.05 h), respectively) and the lack any consistent time dependence in the shifting effect of GA (Figure 1B).

### 2.2. Effects of GA on Rhythms of PERIOD2 Expression in PER2::LUC Mice

**Figure 2:**
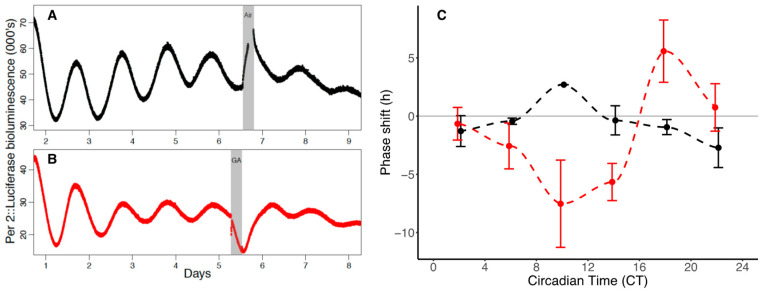
(**A**,**B**) Luminescence recording of SCN slices in culture over the course of eight days. Circadian rhythms in luminescence directly reflect the expression of the PERIOD2-fusion protein in the SCN tissue. Following at least three complete cycles SCNs were exposed to (**A**) a six-hour air (Control) exposure (SCN #26) or (**B**) a six hour 2% isoflurane GA (in air) (SCN #53). Following GA luminescence is suppressed (due in part to the transient competitive binding between anaesthesia luciferin and luciferase). Rhythmic expression of the PER2::LUC resumes and the phase of the rhythms is compared to those prior to GA (see methods). (**C**) Phase response curves for the effect of six hours of GA (2% isoflurane in air) (red) and control (black) on the phase of rhythms of PER2::LUC in the SCN. A profound phase delay portion occurs between CT2 and CT15 and a more modest phase advance section occurs between CT17–22. Filled circles represent the mean in each four-hour time bin (error bars indicate SEM). Circadian Time of GA is defined as the time from onset of activity (CT12) until the midpoint of the six-hour GA. Control values have been slightly shifted (horizontally) for visualization. Dashed lines represent the cubic spline between means.

Of the 89 transgenic C57 PER2::LUC animals euthanised for bilateral SCN preparation and organotypic slice culture, five dissections were unsuccessful resulting in no SCN tissue being transferred into culture. Of the remaining 84 SCNs, a further nine did not display robust rhythms in PER2::LUC expression in culture prior to or after GA and so were excluded from analysis. Data from the remaining 75 SCNs was analysable and used for construction of the phase response curves (PRCs) for “treatment” (six hours of GA n = 38) (Figure 2B,C) and “control” (six hours air (n = 37)) (Figure 2A,C, Table 1) (Supplementary Table S2, Supplementary Figure S2).

As with the behavioural PRC, the shape of the PER2::LUC PRC is a weak (Type One) curve (Figure 2C). However, the magnitude of the phase shifts is some ten times larger. A long and large magnitude delay portion from CT2–CT15 shows maximum phase delay of −7.53 h (±3.75 h) and a relatively short advance portion from CT17–CT22 with a maximum phase advance of 5.58 h (±2.67 h) (Figure 2C). Notable differences between the behavioural and PER2::LUC PRCs (apart from the magnitude of the phase shifts elicited) include the earlier timing of the phase delay portion in the PERIOD2 PRC (which precedes behavioural phase delays by approximately 4 h) and the presence of a substantial advance portion.

## 3. Discussion

Over the past decade, international research efforts have provided incontrovertible evidence that general anaesthetics shift the circadian clock. The implication of this is that, following anaesthesia and surgery, patients’ clocks may be disrupted and this may affect recovery. The somewhat frustrating thing about the existing data is that there is little agreement about how and when these shifting effects occur—one of the few commonalities is a tendency for phase delays to be reported in free-running conditions and advances in entrained conditions [7]. A clear understanding of the time dependence of the effects of GA on the clock is essential to understand how we might go about mitigating the effects of GA on the clock using chronotherapeutic principles.

By conducting methodical experiments at all different circadian phases, we can carefully establish the effects of GA on the circadian systems of various animal models, construct phase response curves to explain their time dependence, and compare these effects directly to the effects of known zeitgebers such as light. A clear chronotherapeutic approach to reducing GA-induced phase-shifting can then be examined.

In 2016, our group produced a PRC for the effects of the inhalational anaesthetic isoflurane (2% for 6 h) on behavioural rhythms in honey bees. In 2020, we published our work describing the phase responses for isoflurane on behaviour and period gene expression in the fruit fly *Drosophila melanogaster* [16] (again 2% for 6 h). Here, we present the first full PRC for the effects of GA with isoflurane on behavioural rhythms in a mammalian model, and the effects of GA of the same concentration and duration on PER2::LUC expression in these mice.

The only comparable study that has conducted anaesthesia at all different circadian phases is a recent paper by Imai et al. [13] examining the inhalational agent desflurane. In their paper, in which they administer subclinical doses of desflurane (4%) to C57 mice, the authors show phase delays in behaviour at all circadian phases apart from CT 5, but a major phase-delay portion in line with our study (between CT10 and CT 20) with a maximum phase shift of −1.7 h.

In contrast to our study, Imai et al., only examine gene expression at a single time-point after GA. Therefore, it was not possible for them to determine the presence or absence or timing of phase shifts in clock gene expression in this instance. The acute effects of GA they report appear to be an increase in expression of *Per2*, *Bmal1* and *Cry1.* This contrasts to the findings presented here, those from *Drosophila* [16], and previous mouse work from other groups [10,11,12].

Our observation that PER2::LUC expression is phase delayed between CT2 and CT14 is consistent with previous work examining the effect of the activation of GABA receptors on *Per1* and *Per2* expression in the SCN of hamsters [10,11]. *Per1* and *Per2* expression is reduced in the SCN of hamsters that were given muscimol (a GABA agonist) at CT6. Furthermore, administration of sevoflurane (2.5%) between ZT2—ZT6 (which equates to c. CT2–6 in our data) is a time at which this agent prevents CLOCK protein from binding to the E-box of *Per2*.

Given the close agreement between our findings and those using GABA acting agents such as muscimol, a parsimonious explanation of the mechanisms underlying the effect of isoflurane of the clock is via its action on GABA. A further clarification of whether this may be the case will rely on the examination of the effect of isoflurane on GABA receptor expression in the SCN, and the effect of non-GABA acting anaesthetics such as xenon and ketamine on clock gene expression and behaviour.

The acute suppression of luminescence from the PER2::LUC SCN organotypic cultures immediately following GA (Figure 2B) has previously been attributed to the competitive inhibition of binding between luciferin and luciferase by the anaesthetic [18]. However, if anaesthesia acts on the clock by preventing the CLOCK-BMAL1 activator from binding to the E-box of *Per2*, then we could predict that some of the suppression of luminescence following GA might be attributable to anaesthesia preventing its activation. If this is the case, one would also predict that *Per2* suppression would only occur when CLOCK was nuclear rather than cytosolic. Certainly, the times at which maximal phase delays of *Per2* expression occur in our data are times at which CLOCK is nuclear. The presence of an advance portion of the *Per2* PRC at late CTs are not explained by effects through CLOCK, however, as they occur at times when CLOCK is cytosolic. The effect of GA on phase shifting is presumably not operating entirely through a CLOCK-Ebox mediated mechanism.

The consistency between the effects of isoflurane GA on mice and flies is quite remarkable, both at the behavioural and clock gene expression level. Isoflurane phase delays mouse behaviour by approximately an hour in the afternoon and evening (between CT6 and CT18) and in flies, a delay of up to an hour also occurs the afternoon and evening/night (between CT10 and CT 22). Mice show a smaller phase advance portion of approximately 15 min (between CT20 and CT2) than *Drosophila* (that phase advance up to an hour between CT24 and CT8).

The similarities in the effects between our vertebrate and invertebrate models are not restricted to the effects of GA on behaviour, however. Mice show large magnitude (up to 7.5 h) delays in PER2 expression between CT 2 and CT14. These effects are some ten times larger than the effects on behaviour, and precede the behavioural shifts by approximately four hours. In flies, effects on period expression in the central clock neurons of the brain also precede those on behaviour (by approximately four hours) and are twice as large as behavioural shifts.

Why such profound differences between the effect of GA on PER2 expression and behaviour are present, remains to be understood. However, reports of stronger effects of clock manipulations in SCN clock gene express in vitro compared to in vivo behavioural effects are present in the literature [19]. A plausible explanation for the difference we have observed may lie in the fact that in vivo GA has the potential to affect not only the SCN but also peripheral oscillatory systems, and that the combined effects of GA on the entire circadian neuroendocrine axis presumably controls behaviour. Equally, the smaller magnitude effects of GA on behaviour compared to PER2 expression could result from a lack of effect of GA on peripheral oscillators. It is well established that peripheral oscillators can feed back onto the SCN clock, and this in turn can affect behaviour. A GA-induced internal desynchrony between the SCN and peripheral oscillators could cause the disruption experienced by patients post-operatively.

In the SCN experiments described here, the effects on gene expression were of course examined ex vivo. An understanding of whether GA may also affect noncentral-clock oscillators will rely on a phase mapping approach in mammals (administering GA in vivo and dissecting central and peripheral tissues to examine the effect on the phase of different tissues), or in *Drosophila*, the use of reporters such as XLG, which report period expression from the entire body rather than just the central clock neurons. It is possible that the lower magnitude of the shifts in period gene expression observed in *Drosophila* compared to the ex vivo SCN might be attributable to the fact that the *Drosophila* experiments were conducted in whole animals rather than isolated tissue. When the mouse and fly responses are compared to those from the honey bee, there are notable differences. The fly *(Drosophila melanogaster)* and the honey bee are, of course, diurnal while the mouse is nocturnal, but at a molecular level the honey bee clock arguably operates in a way more similar to the mammalian clock than *Drosophila.* The effects of GA on the honey bee are, however, unlike either the mouse or the fly. In honey bees, isoflurane delays behavioural rhythms in the subjective morning (between CT0 and CT 9), some six hours earlier than in mice, and a time at which there are profound phase advances in the fly.

When comparing these effects to those of light (the major zeitgeber for the clock) for each of the three species, one can see that the effects of GA and light on the clock are opposing in the honey bee at the same CTs (at least in the morning) [17,20]. In contrast, GA induces phase delays in *Drosophila* and mice at similar CTs despite the fact that *Drosophila* are diurnal and mice are nocturnal. It would be interesting to see the timing of phase shifting effects of GA in a diurnal mammalian model.

Recent efforts to develop approaches to reduce anaesthesia-induced clock shifting have investigated the use of melatonin [14] and 5-HT7 serotonin receptor agonists and antagonists [15] on GA-induced circadian disruption in mice. While melatonin is proposed to reduce anaesthesia and surgical disruption [14] and 5-HT7 serotonin receptor antagonists “alleviate circadian rhythm disorder” in mice, no clear phase responses of these effects have been described.

In terms of chronotherapaeutic approaches to reduce post-operative circadian disruption in patients, if GA affects the clock in humans in a manner similar to that of the diurnal honey bee (with GA phase shifts early in the subjective day when light causes phase advances) the concomitant administration of light during anaesthesia may provide the basis as a valid approach to reducing GA-induced phase delays. However, if GA acts in humans as it does in *Drosophila* and mice (with GA and light-induced phase delays occurring at the same CTs), approaches to reducing GA induced clock shifts are somewhat different—the elucidation of this will rely on results of clinical studies.

## 4. Materials and Methods

### 4.1. Behavioural Experiments

All procedures described were approved by the University of Auckland Animal Ethics Committee (approval # 001654/2 & 001128 on 3/12/2015 and 12/12/2014). Eight to twelve week old male C57BL/6 mice were supplied by the Vernon Jansen Unit at the University of Auckland. C57BL/6VJU mice show robust and persistent circadian rhythms in wheel running behaviour and are the background of the transgenic PER2::LUC reporter line used in the SCN work described below.

Animals were housed individually in standard transparent cages fitted with a rodent running wheel attached to an infrared microswitch (Actimetrics, Wilmette, IL, USA). Running wheel activity was recorded for circadian analysis using the Clocklab system (Actimetrics, IL, USA). Cages were maintained in ventilated, sound attenuated, temperature-controlled environmental chambers for the duration of the experiments at 23 °C ± 1 °C.

In the behavioural experiments, locomotor activity rhythms of individual animals were recorded for a period of 40 days. Each animal served as its own control, receiving a 10 min “control” anaesthetic or a 6 h “treatment” anaesthetic using the following protocol: after 10 days of light cycles (LD 12:12) animals were transferred into constant darkness (DD) for 14 days prior to their treatment (either 6 h or 10 min determined in a randomized order). Their activity was then monitored for a further 14 days, and on the subsequent (15th) day they received the other arm of their treatment, after which behaviour was recorded for a final 14 days prior to the end of the experiment and euthanasia. As the mouse circadian clock is exquisitely light sensitive, during DD all procedures (including GA) were conducted in complete darkness using night vision goggles.

In all cases, GA with isoflurane (6 h “treatment” or 10 min “control”—in which control animals were anaesthetized for 10 min and then returned to their cages) was conducted in an anaesthetic chamber in 100% oxygen. Heating pads were used to maintain body temperature, and soda lime in the base of the chamber was used to absorb excess carbon dioxide. Anaesthetic vapour was supplied by using a standard isoflurane vaporizer (MSS, Yorkshire, UK) attached to medical oxygen supply (BOC, Auckland, New Zealand) administered in a flow through system with the outlet from the chamber attached to an activated charcoal scavenger (Cardiff Aldasorber, Cardiff, UK).

Anaesthetic induction with 5% isoflurane (4 L/min) typically occurred within 10–30 s, after which the anaesthetic concentration was reduced and maintained between 1.5% and 2% (1 L/min) for the duration of the treatment. Anaesthesia was deemed to occur when animals showed a loss of righting reflex and no response to tail pinch. Spontaneous respiratory rate was monitored (visually) every five minutes and maintained above 30 breaths/min at all times. In the event that respiratory rate dropped below 30, animals were placed in a second oxygen chamber (without isoflurane) until respiratory rate had recovered, at which point they were returned to the anaesthetic chamber. Using this protocol mortality rate from anaesthesia was zero. After five and a half hours of GA the vapourizor was switched off, and animals were partially recovered in the chamber and then returned to their individual cages prior to emergence. Emergence time (as determined by return of righting reflex) varied between individuals but typically took 20–25 min. Anaesthetic concentrations of the outflow from the anaesthetic chamber were monitored using a Datex-Ohmeda Cardiocap 5 gas analyser (GE Healthcare, Auckland, New Zealand).

### 4.2. Behavioural Analysis

Circadian times of treatment were calculated using CT12 (onset of activity using the Clocklab function using the 20th percentile of activity levels (https://www.harvardapparatus.com/media/manuals/Product%20Manuals/ACT-500%20ClockLab%20Analysis%20Manual.pdf)) as a phase reference point, and the midpoint of the GA as the time at which GA occurred. Free-running periods of behavioural rhythms were calculated prior to and after GA using the χ^2^ periodogram analysis function in Clocklab. Shifts in the phase of behavioural rhythms were calculated (using onset of activity as the phase reference point and a least squares fit through the onsets) on the seven days prior to and following the administration of GA.

### 4.3. Organotypic SCN PERIOD2::LUC Gene Expression Experiments

The transgenic PER2::LUC mice used in these experiments are well described elsewhere [21,22]. This fusion protein reporter line co-expresses luciferase with the clock protein PER2. Thus, levels of PER2 protein in a tissue can be inferred by recording the bioluminescence of these tissues. Organotypic SCN slices can be maintained in culture for extended periods of time (days–months) and PER2 expression faithfully reported from the SCN by luciferase signalling.

Five founder homozygous PER2::LUC knockin breeding pairs of mice (B6.*129S60Per2^tm1Jt^/*J) imported from the Jackson Laboratory (JAX) U.S.A (MPI Import permit # 2014052922) were crossed with C57BL/6VJU and maintained as a colony of heterozygous animals at the University of Auckland. As described by [21], heterozygous 8–12 weeks old mice were euthanized by cervical dislocation and decapitated between ZT2 and 3 (10:00–11:00 am). Dissected brains were rapidly cooled to 4 °C in Hank’s balanced salt solution (HBSS), mounted, and sliced (300 μm) until the SCN was isolated. Bilateral SCN tissue was excised and cultured on millicell culture inserts (hydrophilic PTFE 0.4 μm) (Sigma Aldrich, Auckland, New Zealand) in a 35 mm petri dish containing 1 mL of warmed luciferin-fortified media.

Petri dishes containing SCNs were placed into custom-made borosilicate glass anaesthetic chambers (sealed with vacuum grease) inside a light-tight recording box, which itself was placed in a Percival environmental cabinet (I36-NL) maintained at 35 °C. Four SCN cultures were placed within each recording box beneath a highly sensitive photomultiplier tube (Hammamatsu Photon Counting Head H9313 dark count 50 (below 50 counts/second at room temperature), Hammamatsu, Japan) powered by a 5 volt supply and connected to a data recording computer (via USB-RS232 data cables). Data was collected using a custom-made recording program (PMT-MON version 1.5-060804).

Following five days of baseline recordings of PER2::LUC oscillations, SCNs were exposed to a six-hour isoflurane “treatment” (2%) (or a six-hour air “control”) at a flow rate of 1 L/min. Post-GA recordings were continued for a further five days after treatment.

As above, anaesthetic vapour was supplied using a standard isoflurane vapourizer (MSS, Yorkshire, UK), attached to an air supply in a flow through system with the outlet from the chambers attached to an activated charcoal scavenger (Cardiff Aldasorber, UK). In addition, however, anaesthetic vapour was humidified prior to introduction into the culture systems using a Fisher and Paykel Humiguard MR860AE (Fisher and Paykel Helathcare, Auckland, New Zealand). Anaesthetic concentration at the outflow from the anaesthetic chamber was monitored using a Datex-Ohmeda Cardiocap 5 gas analyser (GE Healthcare, New Zealand).

### 4.4. Organotypic SCN PER2::LUC Data Analysis

**Figure 3:**
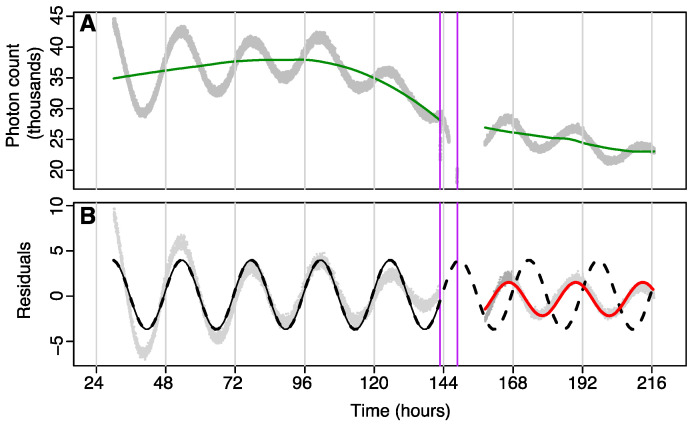
(**A**) Separate high-pass filters (green lines) removed general trends from data (grey points) to give residuals. Treatment start and end times (either GA or air) are shown as vertical purple lines. (**B**) The cosinor model fit to pre-treatment residuals (solid black line) was forecast into post-treatment time and compared with a cosinor model of the post-treatment data (red line). The phase change (*Φ*) was determined by the difference between the first acrophase and the closest forecast acrophase. Raw data during GA and immediately following GA were removed prior to analysis (as can be seen in panel **A**) as luciferase expression is directly inhibited by GA during these times (see Figure 2B) and inclusion of this artefactual data would confound the curve fitting of the post-GA data.

Data were analysed using a cosinor model fit to *tau* before and after the administration of isoflurane or control, and phase shifts determined using acrophase on the first post-treatment day. Data were split into two parts: a pre-treatment section consisting of all data (i.e., recorded photon counts) prior to treatment, and a post-treatment section, which used data from the point where rhythmicity was considered to have re-established (see Figure 3B) to the end of recording. Pre- and post-treatment data were passed through separate high-pass filters to remove multiday trends due to consumption of luciferin in the media using a loess smoother with tri-cubic weighting and a bandwidth spanning 90% of the data (Figure 3A shows an example). The residuals were then analysed using two separate cosinor models. One model was fit to just pre-treatment data, and the second model was fit to post-treatment data. The second model used data starting from the point where rhythmicity was considered to have re-established (see Figure 3B). The pre-treatment model was then extrapolated into the post-treatment times, and the phase-shift was calculated as the change in acrophase (in hours) on the first post-treatment day (this was done since pre-treatment and post-treatment models could have different periods). Time at which GA occurred was calculated using the midpoint of GA as the phase reference point.

Mean data binned in four-hour epochs (CT0–4, CT4–8, CT8–12, CT12–16, CT16–24) were plotted as phase response curves (±SEM).

### 4.5. Statistical Analysis

#### 4.5.1. Comparing GA vs. Control

Differences between the average control and GA phase shifts were examined using a permutation test for each of those PRC time-points with a combined sample size (i.e., GA and control) greater than twelve. At each of these PRCs times, the permutation test randomly permuted groups’ labels and recorded the absolute difference between the permuted group means. This was replicated 1999 times to create a null distribution. The absolute value of the observed difference was then compared to the null distribution to determine a (2-sided) *p*-value.

#### 4.5.2. Testing Whether Controls Differed from Zero

Non-zero differences in median phase shift for the control at each binned PRC time point were examined using the Wilcoxon Signed rank test with continuity correction.

## Figures and Tables

**Table 1:** Summary statistics of Circadian Times and phase shifts for behavioural and PER2::LUC phase shifting experiments. Phase shifts of general anaesthesia (GA) bins were tested for statistical significance from controls using permutation tests. Differences in controls from zero were tested using Wilcoxon Signed rank test. Evidence of differences from control at the *p* < 0.01 level are indicated with two asterisks and at *p* < 0.05 level with one asterisk.

		Control		GA	
Behavioural	CT (range)	Sample Size	Shift (SE) (h)	Sample Size	Shift (SE) (h)
	2 (0.5–2.4)	7	−0.07 (0.08)	19	0.17 (0.14)
	6 (4.3–7.6)	9	0.02 (0.16)	7	−0.11 (0.38)
	10 (8.2–11.8)	8	0.06 (0.24)	8	−0.61 (0.31)
	14 (12.4–15.9)	7	0.06 (0.12)	8	−0.77 (0.18) **
	18 (16–20)	23	−0.14 (0.05) **	21	−0.25 (0.11)
	22 (20–24)	24	−0.12 (0.04) **	17	0.23 (0.14) **
**PER2::LUC**					
	2 (0–4.0)	9	−1.29 (1.32)	9	−0.66 (1.40)
	6 (4.9–7.1)	3	−0.44 (0.27)	4	−2.57 (1.96)
	10 (9.5)	1	2.70 (-)	2	−7.53 (3.75)
	14 (12.2–15.8)	8	−0.37 (1.25)	13	−5.65 (1.59) *
	18 (16.3–19.2)	9	−0.95 (0.65)	3	5.58 (2.67) *
	22 (20.9–22.4)	7	−2.71 (1.70)	7	0.75 (2.04)

## Supplementary Materials

The following are available online at https://www.mdpi.com/2624-5175/3/1/6/s1, Figure S1: Behavioural actograms of 80 C57BL6/VJU mice exposed to control (10 min) GA, or treatment (6 h) GA with isoflurane. Figure S2: SCN PER2::LUC Expression Experimental Data. Table S1: Table of Behavioural Phase shifts of C57 BL6/VJU mice exposed to isoflurane GA (Control = 10 min, GA= 6 h). Table S2: Table of SCN PER2::LUC shifts exposed to isoflurane GA.
