# Supplementary material for: General Anaesthesia Shifts the Murine Circadian Clock in a Time-Dependant Fashion
Source: Clocks Sleep. 2021 Jan 26;3(1):87–97. doi: 10.3390/clockssleep3010006 (PMC7930986; doi:10.3390/clockssleep3010006)

Supplementary Materials:

Figure S1: Behavioural actograms of 80 C57BL6/VJU mice exposed to control (10 min) GA, or treatment (6 hour) GA with isoflurane. Yellow blocks indicate timing of the light portion of the LD 12:12 cycle and red bars indicate the GA.

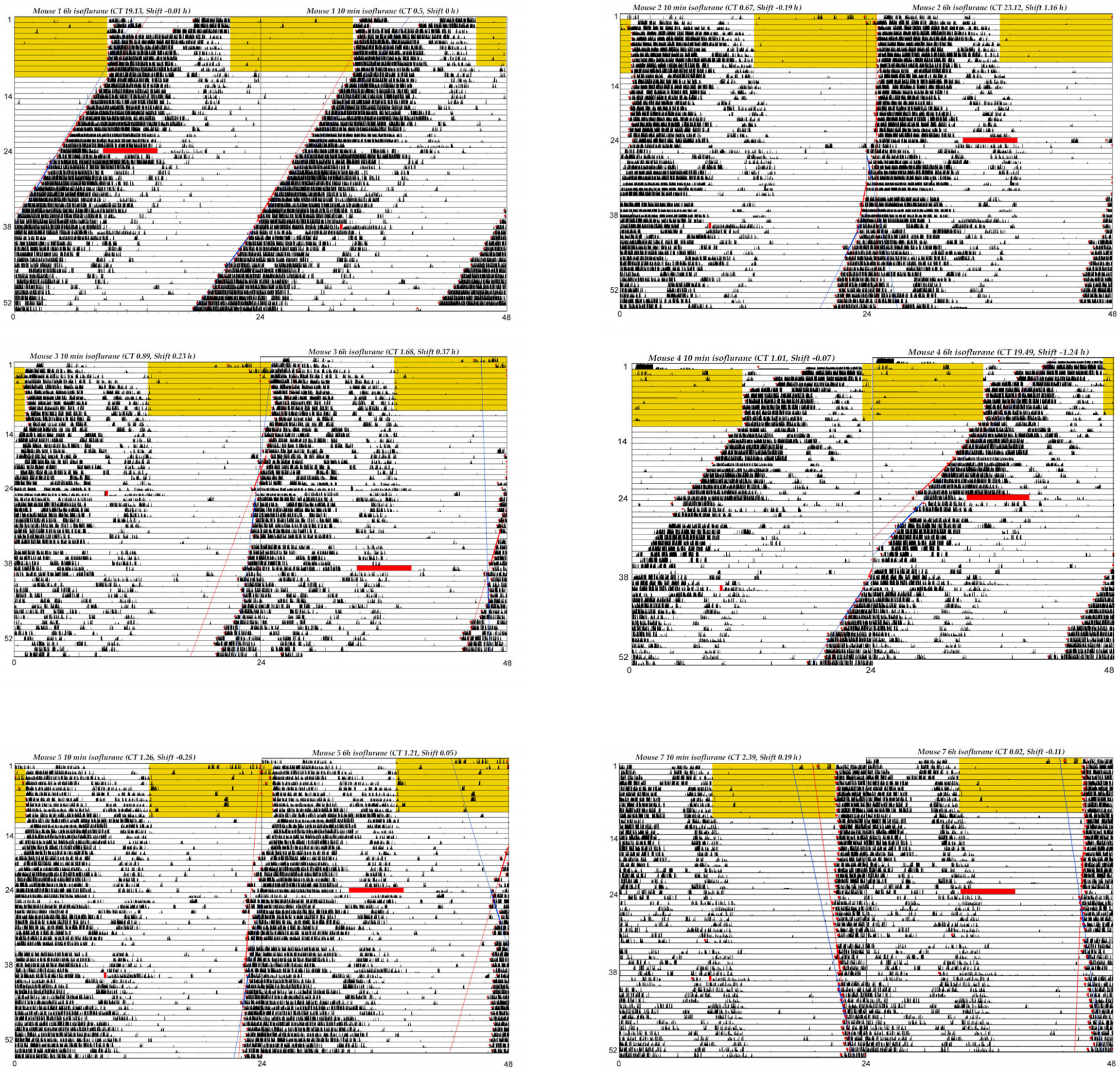

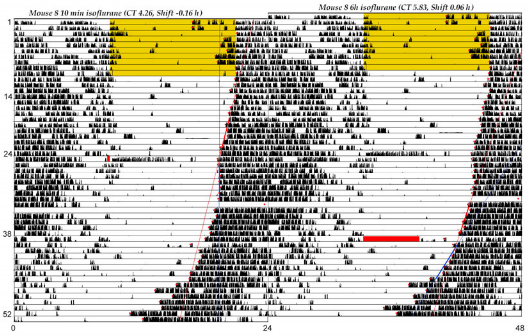
Mouse 8 10 min isoflurane (CT 4.26, Shift -0.16 h)
Mouse 8 6h isoflurane (CT 5.83, Shift 0.06 h)
1
14
24
38
52
0
24
48

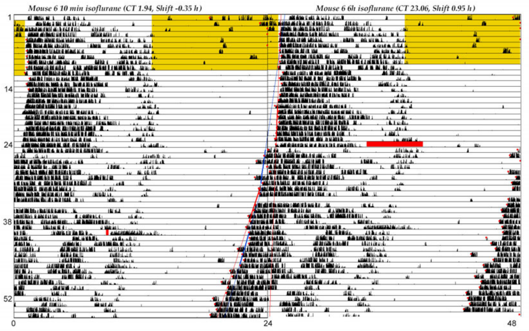
Mouse 6 10 min isoflurane (CT 1.94, Shift -0.35 h)
Mouse 6 6h isoflurane (CT 23.06, Shift 0.95 h)
1
14
24
38
52
0
24
48

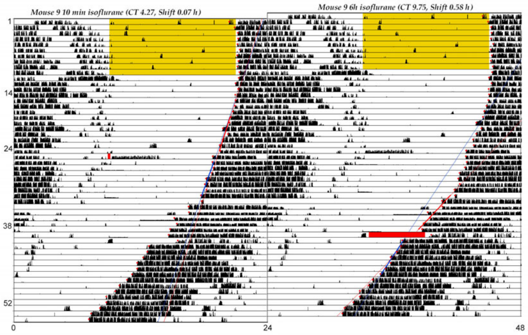
Mouse 9 10 min isoflurane (CT 4.27, Shift 0.07 h)
Mouse 9 6h isoflurane (CT 9.75, Shift 0.58 h)
1
14
24
38
52
0
24
48

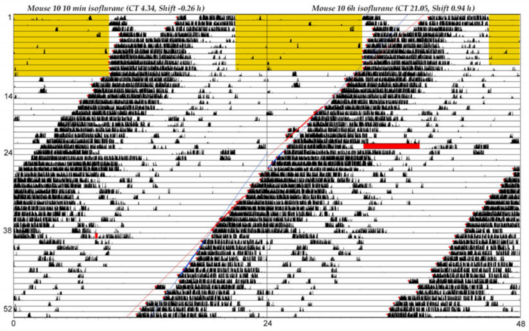
Mouse 10 10 min isoflurane (CT 4.34, Shift -0.26 h)
Mouse 10 6h isoflurane (CT 21.05, Shift 0.94 h)
1
14
24
38
52
0
24
48

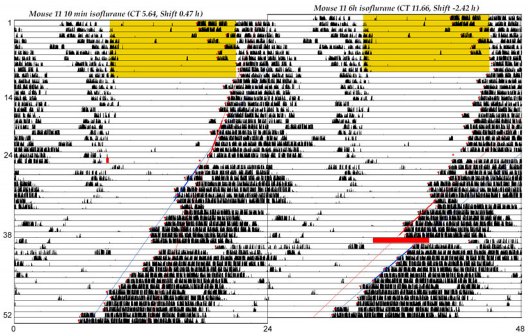
Mouse 11 10 min isoflurane (CT 5.64, Shift 0.47 h)
Mouse 11 6h isoflurane (CT 11.66, Shift -2.42 h)
1
14
24
38
52
0
24
48

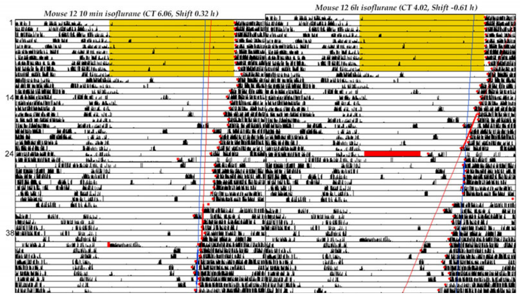
Mouse 12 10 min isoflurane (CT 6.06, Shift 0.32 h)
Mouse 12 6h isoflurane (CT 4.02, Shift -0.61 h)
1
14
24
38

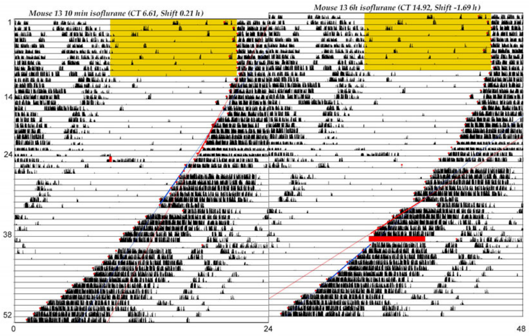
Mouse 13 10 min isoflurane (CT 6.61, Shift 0.21 h)
Mouse 13 6h isoflurane (CT 14.92, Shift -1.69 h)
1
14
24
38
52
0
24
48

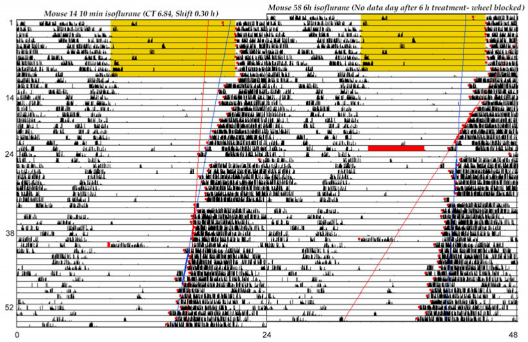
Mouse 14 10 min isoflurane (CT 6.84, Shift 0.30 h)
Mouse 58 6h isoflurane (No data day after 6 h treatment- wheel blocked)
1
14
24
38
52
0
24
48

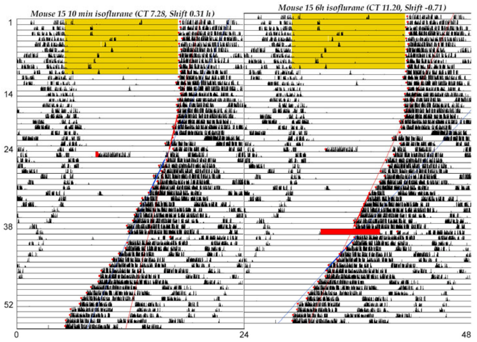
Mouse 15 10 min isoflurane (CT 7.28, Shift 0.31 h)
Mouse 15 6h isoflurane (CT 11.20, Shift -0.71)
1
14
24
38
52
0
24
48

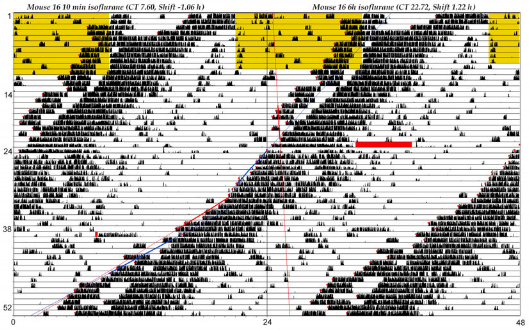
Mouse 16 10 min isoflurane (CT 7.60, Shift -1.06 h)
Mouse 16 6h isoflurane (CT 22.72, Shift 1.22 h)
1
14
24
38
52
0
24
48

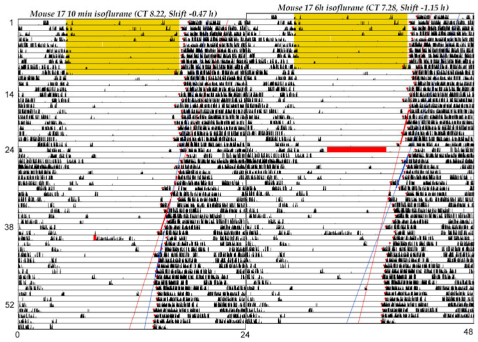
Mouse 17 10 min isoflurane (CT 8.22, Shift -0.47 h)
Mouse 17 6h isoflurane (CT 7.28, Shift -1.15 h)
1
14
24
38
52
0
24
48

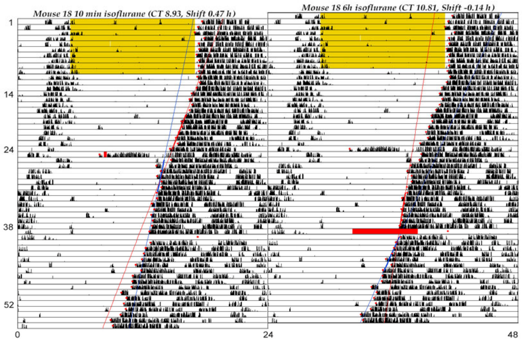
Mouse 18 10 min isoflurane (CT 8.93, Shift 0.47 h)
Mouse 18 6h isoflurane (CT 10.81, Shift -0.14 h)
1
14
24
38
52
0
24
48

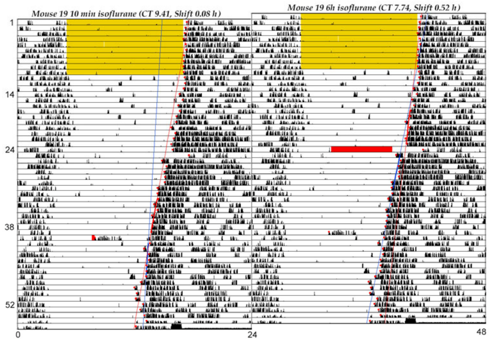
Mouse 19 10 min isoflurane (CT 9.41, Shift 0.08 h)
Mouse 19 6h isoflurane (CT 7.74, Shift 0.52 h)
1
14
24
38
52
0
24
48

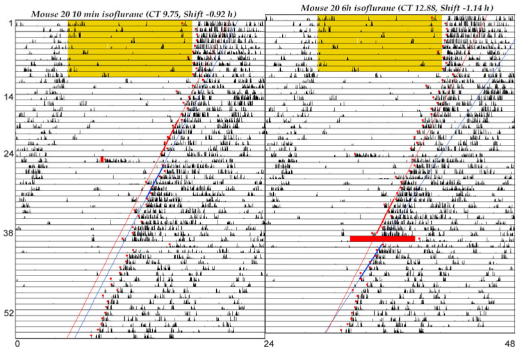
Mouse 20 10 min isoflurane (CT 9.75, Shift -0.92 h)
Mouse 20 6h isoflurane (CT 12.88, Shift -1.14 h)
1
14
24
38
52
0
24
48

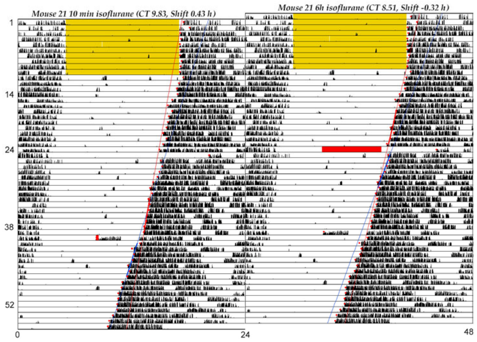
Mouse 21 10 min isoflurane (CT 9.83, Shift 0.43 h)
Mouse 21 6h isoflurane (CT 8.51, Shift -0.32 h)
1
14
24
38
52
0
24
48

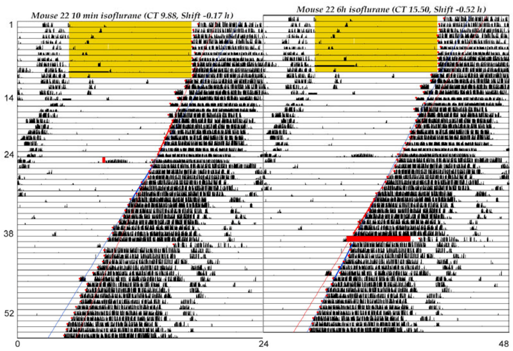
Mouse 22 10 min isoflurane (CT 9.88, Shift -0.17 h)
Mouse 22 6h isoflurane (CT 15.50, Shift -0.52 h)
1
14
24
38
52
0
24
48

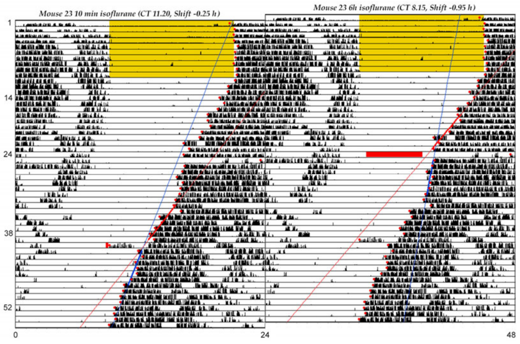
Mouse 23 10 min isoflurane (CT 11.20, Shift -0.25 h)
Mouse 23 6h isoflurane (CT 8.15, Shift -0.95 h)
1
14
24
38
52
0
24
48

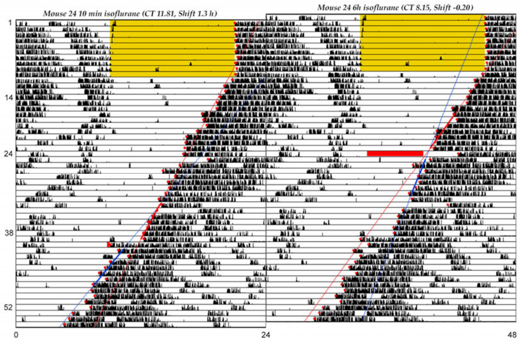
Mouse 24 10 min isoflurane (CT 11.81, Shift 1.3 h)
Mouse 24 6h isoflurane (CT 8.15, Shift -0.20)
1
14
24
38
52
0
24
48

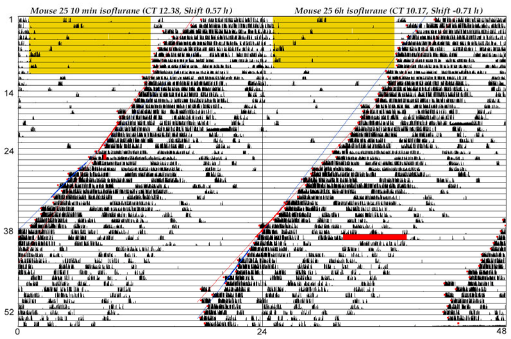
Mouse 25 10 min isoflurane (CT 12.38, Shift 0.57 h)
Mouse 25 6h isoflurane (CT 10.17, Shift -0.71 h)
1
14
24
38
52
0
24
48

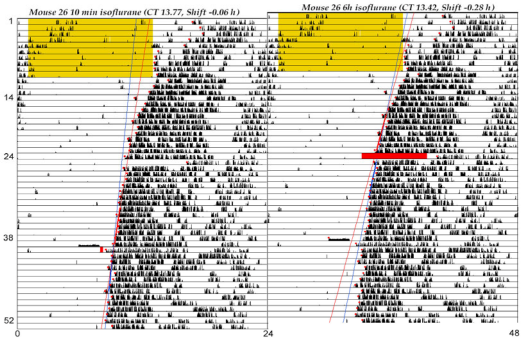
Mouse 26 10 min isoflurane (CT 13.77, Shift -0.06 h)
Mouse 26 6h isoflurane (CT 13.42, Shift -0.28 h)
1
14
24
38
52
0
24
48

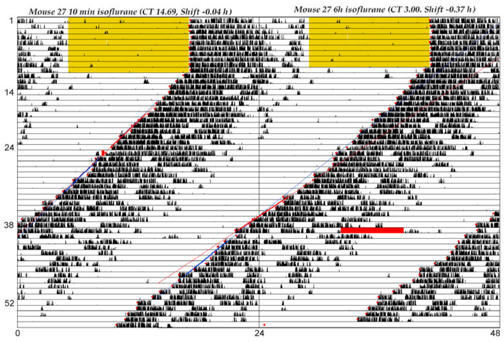
Mouse 27 10 min isoflurane (CT 14.69, Shift -0.04 h)
Mouse 27 6h isoflurane (CT 3.00. Shift -0.37 h)
1
14
24
38
52
0
24
48

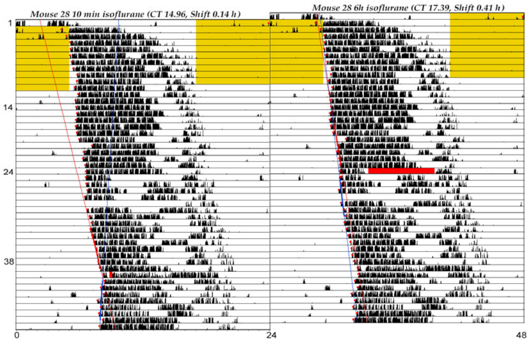
Mouse 28 10 min isoflurane (CT 14.96, Shift 0.14 h)
Mouse 28 6h isoflurane (CT 17.39, Shift 0.41 h)
1
14
24
38
0
24
48

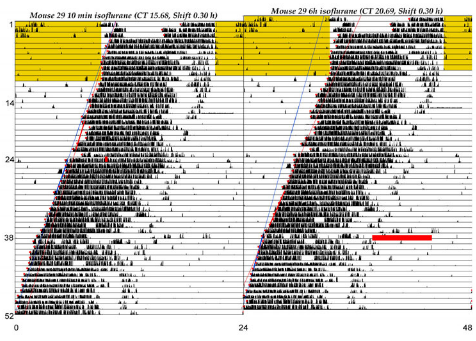
Mouse 29 10 min isoflurane (CT 15.68, Shift 0.30 h)
Mouse 29 6h isoflurane (CT 20.69, Shift 0.30 h)
1
14
24
38
52
0
24
48

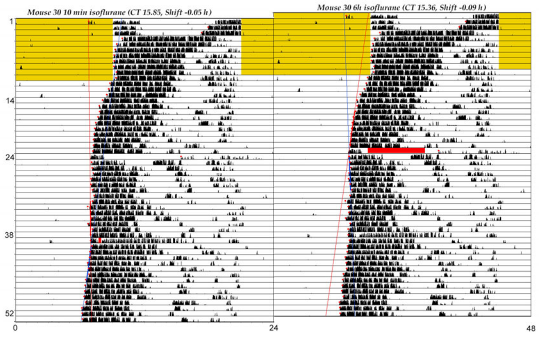
Mouse 30 10 min isoflurane (CT 15.85, Shift -0.05 h)
Mouse 30 6h isoflurane (CT 15.36, Shift -0.09 h)
1
14
24
38
52
0
24
48

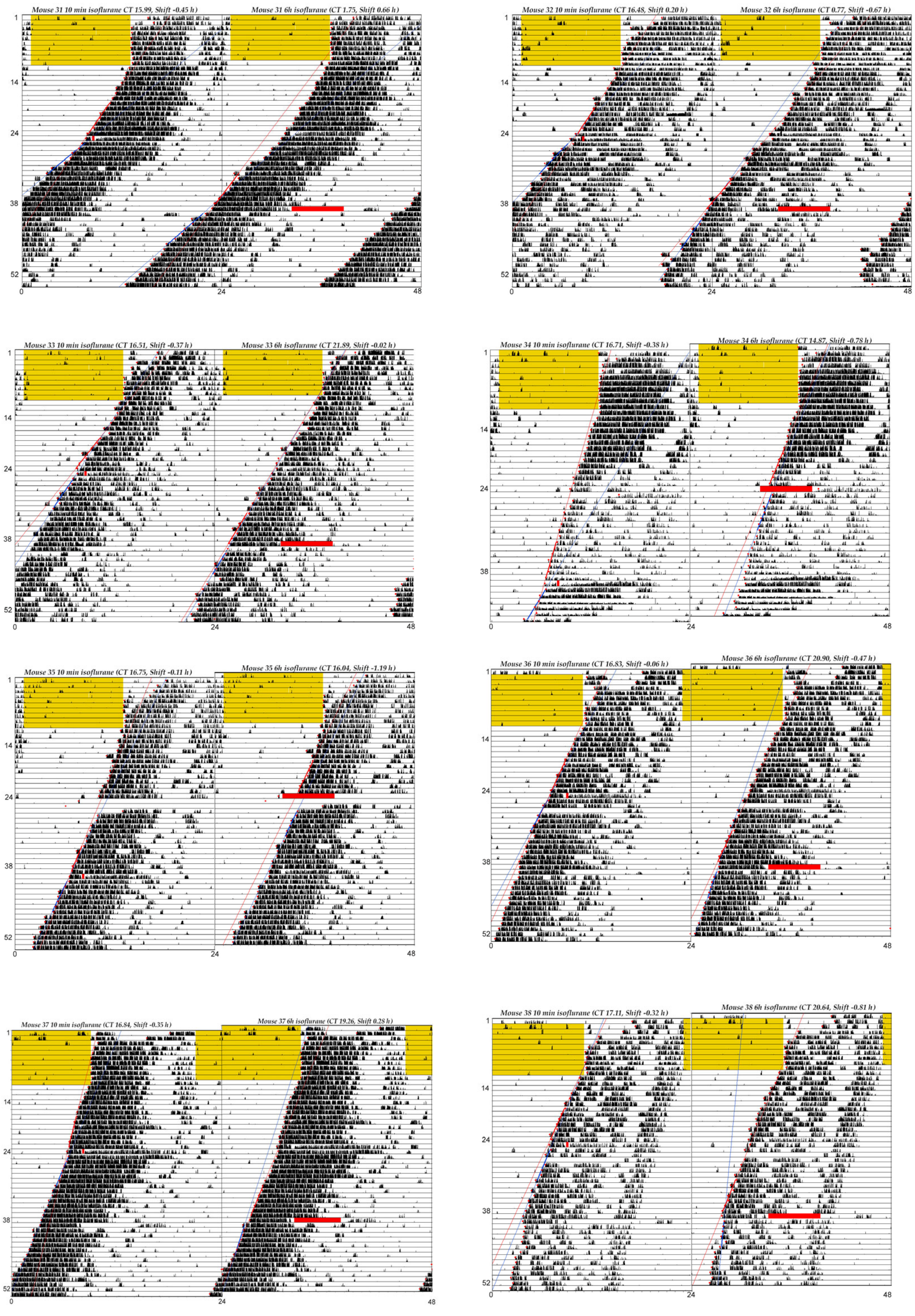

Mouse 31 10 min isoflurane CT 15.99, Shift -0.45 h)
Mouse 31 6h isoflurane (CT 1.75, Shift 0.66 h)
Mouse 32 10 min isoflurane (CT 16.48, Shift 0.20 h)
Mouse 32 6h isoflurane (CT 0.77, Shift -0.67 h)
Mouse 33 10 min isoflurane (CT 16.51, Shift -0.37 h)
Mouse 33 6h isoflurane (CT 21.89, Shift -0.02 h)
Mouse 34 10 min isoflurane (CT 16.71, Shift -0.38 h)
Mouse 34 6h isoflurane (CT 14.87, Shift -0.78 h)
Mouse 35 10 min isoflurane (CT 16.75, Shift -0.11 h)
Mouse 35 6h isoflurane (CT 16.04, Shift -1.19 h)
Mouse 36 10 min isoflurane (CT 16.83, Shift -0.06 h)
Mouse 36 6h isoflurane (CT 20.90, Shift -0.47 h)
Mouse 37 10 min isoflurane (CT 16.84, Shift -0.35 h)
Mouse 37 6h isoflurane (CT 19.26, Shift 0.28 h)
Mouse 38 10 min isoflurane (CT 17.11, Shift -0.32 h)
Mouse 38 6h isoflurane (CT 20.64, Shift -0.81 h)

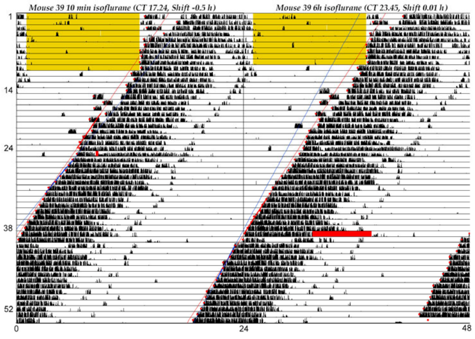
Mouse 39 10 min isoflurane (CT 17.24, Shift -0.5 h)
Mouse 39 6h isoflurane (CT 23.45, Shift 0.01 h)
1
14
24
38
52
0
24
48

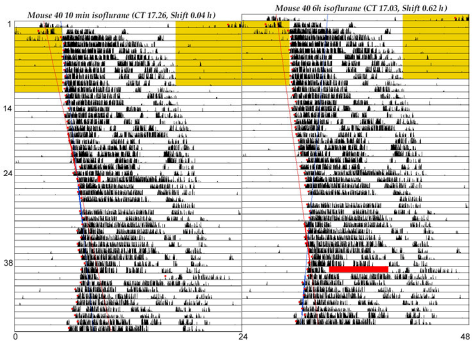
Mouse 40 10 min isoflurane (CT 17.26, Shift 0.04 h)
Mouse 40 6h isoflurane (CT 17.03, Shift 0.62 h)
1
14
24
38
0
24
48

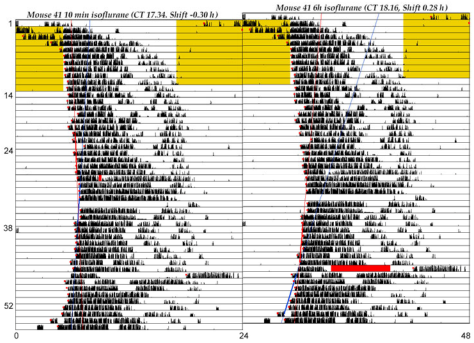
Mouse 41 10 min isoflurane (CT 17.34. Shift -0.30 h)
Mouse 41 6h isoflurane (CT 18.16, Shift 0.28 h)
1
14
24
38
52
0
24
48

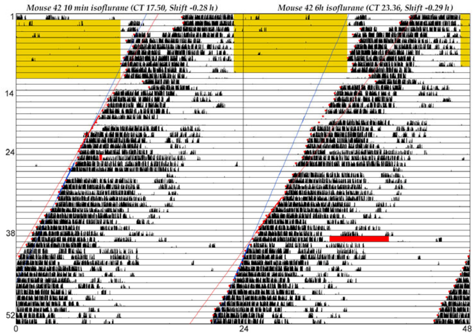
Mouse 42 10 min isoflurane (CT 17.50, Shift -0.28 h)
Mouse 42 6h isoflurane (CT 23.36, Shift -0.29 h)
1
14
24
38
52
0
24
48

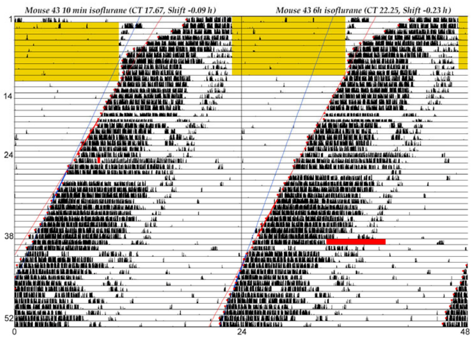
Mouse 43 10 min isoflurane (CT 17.67, Shift -0.09 h)
Mouse 43 6h isoflurane (CT 22.25, Shift -0.23 h)
1
14
24
38
52
0
24
48

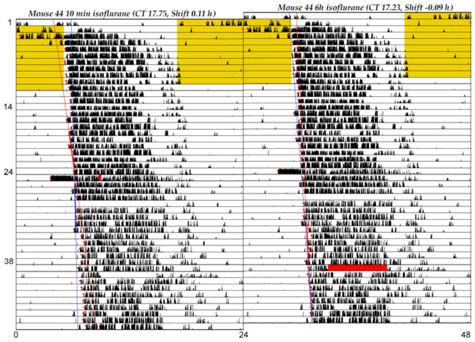
Mouse 44 10 min isoflurane (CT 17.75, Shift 0.11 h)
Mouse 44 6h isoflurane (CT 17.23, Shift -0.09 h)
1
14
24
38
0
24
48

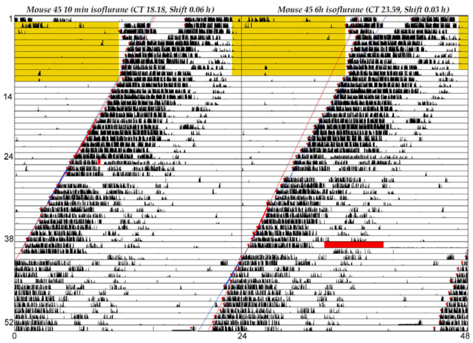
Mouse 45 10 min isoflurane (CT 18.18, Shift 0.06 h)
Mouse 45 6h isoflurane (CT 23.59, Shift 0.03 h)
1
14
24
38
52
0
24
48

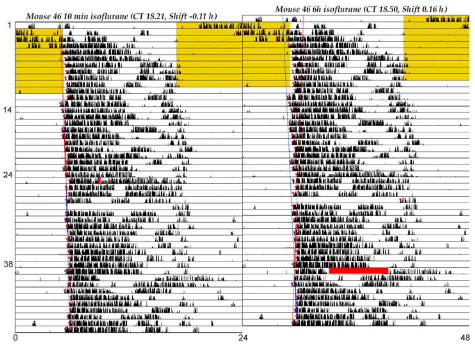
Mouse 46 10 min isoflurane (CT 18.21, Shift -0.11 h)
Mouse 46 6h isoflurane (CT 18.50, Shift 0.16 h)
1
14
24
38
0
24
48

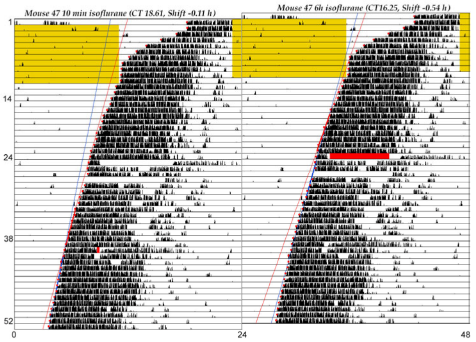
Mouse 47 10 min isoflurane (CT 18.61, Shift -0.11 h)
Mouse 47 6h isoflurane (CT16.25, Shift -0.54 h)
1
14
24
38
52
0
24
48

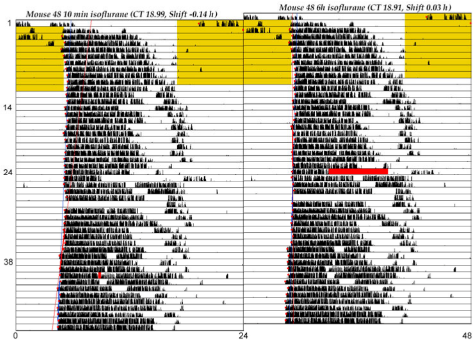
Mouse 48 10 min isoflurane (CT 18.99, Shift -0.14 h)
Mouse 48 6h isoflurane (CT 18.91, Shift 0.03 h)
1
14
24
38
0
24
48

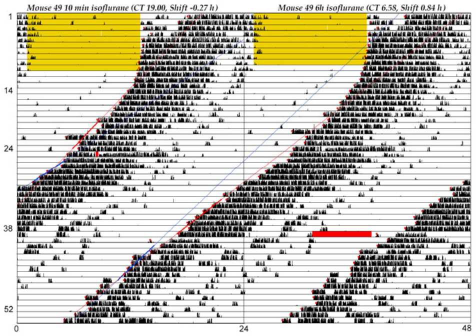
Mouse 49 10 min isoflurane (CT 19.00, Shift -0.27 h)
Mouse 49 6h isoflurane (CT 6.58, Shift 0.84 h)
14
24
38
52
0
24
48

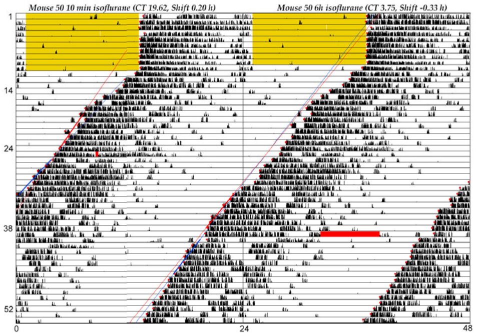
Mouse 50 10 min isoflurane (CT 19.62, Shift 0.20 h)
Mouse 50 6h isoflurane (CT 3.75, Shift -0.33 h)
1
14
24
38
52
0
24
48

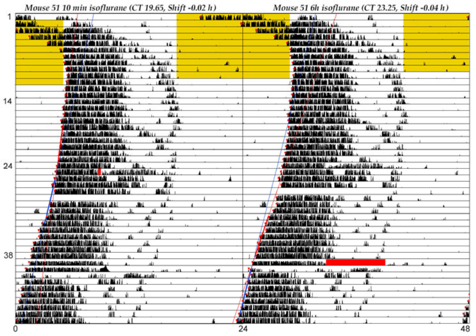
Mouse 51 10 min isoflurane (CT 19.65, Shift -0.02 h)
Mouse 51 6h isoflurane (CT 23.25, Shift -0.04 h)
1
14
24
38
0
24
48

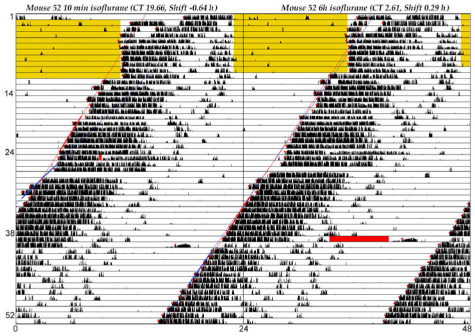
Mouse 52 10 min isoflurane (CT 19.66, Shift -0.64 h)
Mouse 52 6h isoflurane (CT 2.61, Shift 0.29 h)
1
14
24
38
52
0
24
48

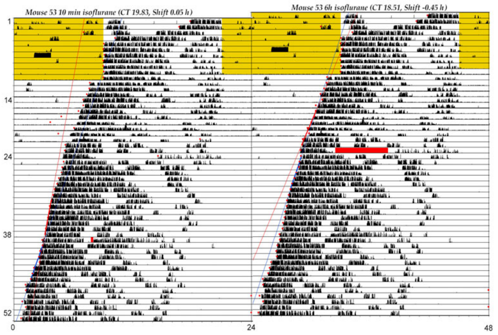
Mouse 53 10 min isoflurane (CT 19.83, Shift 0.05 h)
Mouse 53 6h isoflurane (CT 18.51, Shift -0.45 h)
1
14
24
38
52
0
24
48

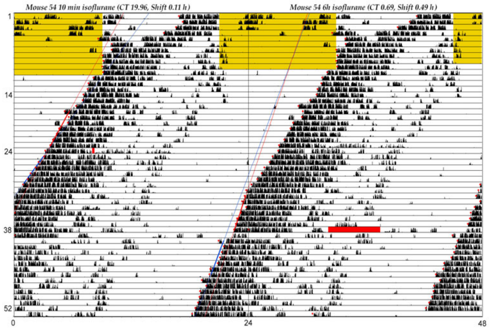
Mouse 54 10 min isoflurane (CT 19.96, Shift 0.11 h)
Mouse 54 6h isoflurane (CT 0.69, Shift 0.49 h)
1
14
24
38
52
0
24
48

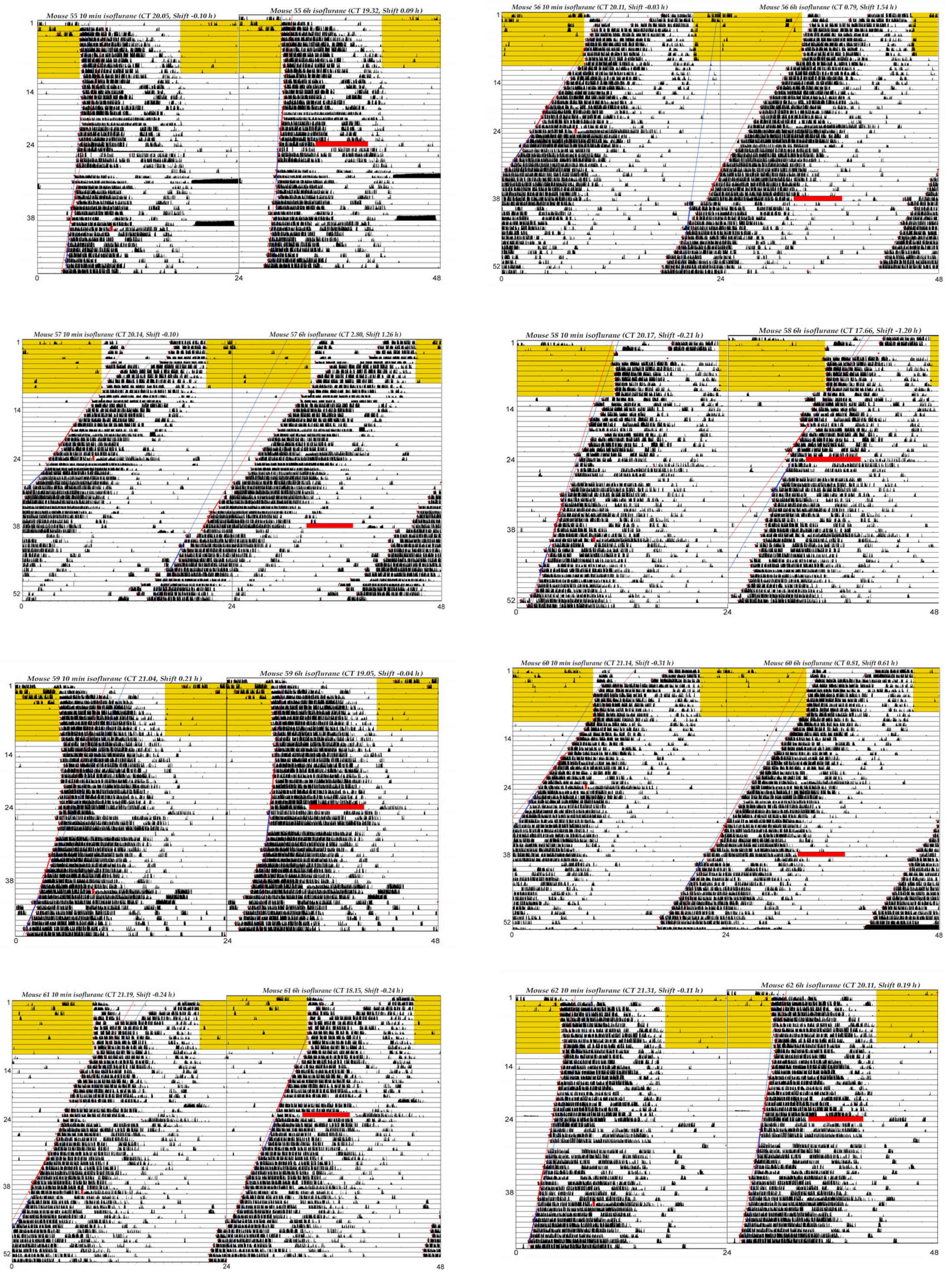
Mouse 55 10 min isoflurane (CT 20.05, Shift -0.10 h)
Mouse 55 6h isoflurane (CT 19.32, Shift 0.09 h)
Mouse 56 10 min isoflurane (CT 20.11, Shift -0.03 h)
Mouse 56 6h isoflurane (CT 0.79, Shift 1.54 h)
Mouse 57 10 min isoflurane (CT 20.14, Shift -0.10)
Mouse 57 6h isoflurane (CT 2.80, Shift 1.26 h)
Mouse 58 10 min isoflurane (CT 20.17, Shift -0.21 h)
Mouse 58 6h isoflurane (CT 17.66, Shift -1.20 h)
Mouse 59 10 min isoflurane (CT 21.04, Shift 0.21 h)
Mouse 59 6h isoflurane (CT 19.05, Shift -0.04 h)
Mouse 60 10 min isoflurane (CT 21.14, Shift -0.31 h)
Mouse 60 6h isoflurane (CT 0.81, Shift 0.61 h)
Mouse 61 10 min isoflurane (CT 21.19, Shift -0.24 h)
Mouse 61 6h isoflurane (CT 18.15, Shift -0.24 h)
Mouse 62 10 min isoflurane (CT 21.31, Shift -0.11 h)
Mouse 62 6h isoflurane (CT 20.11, Shift 0.19 h)

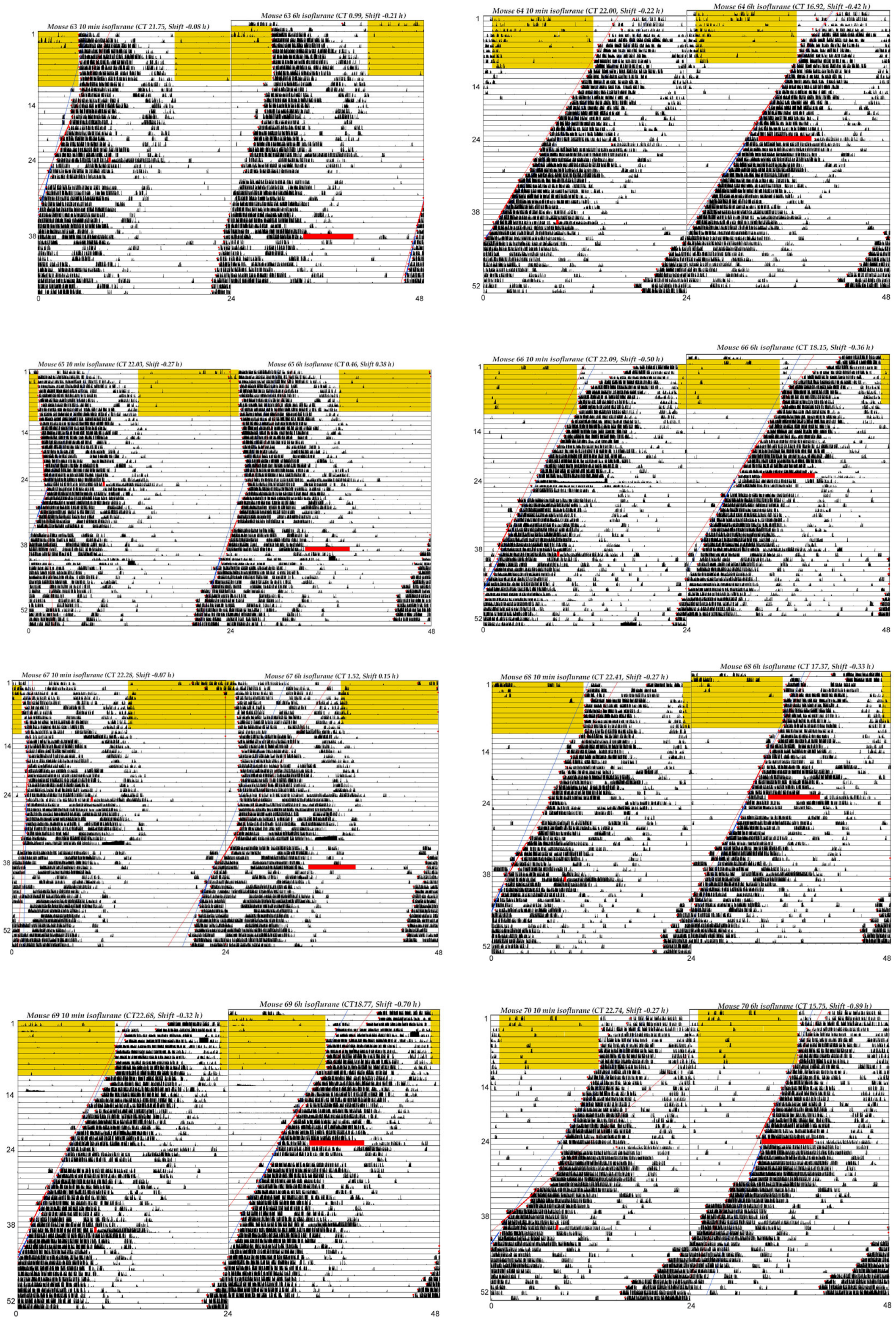

Mouse 63 10 min isoflurane (CT 21.75, Shift -0.08 h)
Mouse 63 6h isoflurane (CT 0.99, Shift -0.21 h)
Mouse 64 10 min isoflurane (CT 22.00, Shift -0.22 h)
Mouse 64 6h isoflurane (CT 16.92, Shift -0.42 h)
Mouse 65 10 min isoflurane (CT 22.03, Shift -0.27 h)
Mouse 65 6h isoflurane (CT 0.46, Shift 0.38 h)
Mouse 66 10 min isoflurane (CT 22.09, Shift -0.50 h)
Mouse 66 6h isoflurane (CT 18.15, Shift -0.36 h)
Mouse 67 10 min isoflurane (CT 22.28, Shift -0.07 h)
Mouse 67 6h isoflurane (CT 1.52, Shift 0.15 h)
Mouse 68 10 min isoflurane (CT 22.41, Shift -0.27 h)
Mouse 68 6h isoflurane (CT 17.37, Shift -0.33 h)
Mouse 69 10 min isoflurane (CT22.68, Shift -0.32 h)
Mouse 69 6h isoflurane (CT18.77, Shift -0.70 h)
Mouse 70 10 min isoflurane (CT 22.74, Shift -0.27 h)
Mouse 70 6h isoflurane (CT 15.75, Shift -0.89 h)

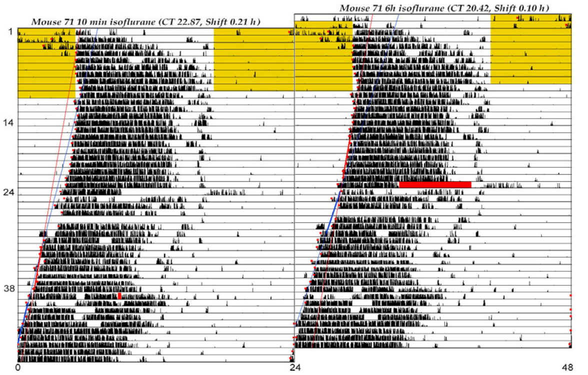
Mouse 71 10 min isoflurane (CT 22.87, Shift 0.21 h)
Mouse 71 6h isoflurane (CT 20.42, Shift 0.10 h)
1
14
24
38
0
24
48

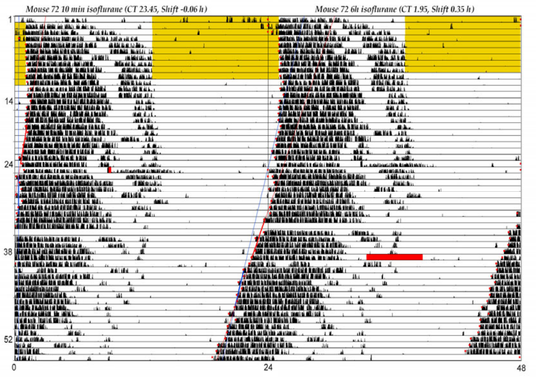
Mouse 72 10 min isoflurane (CT 23.45, Shift -0.06 h)
Mouse 72 6h isoflurane (CT 1.95, Shift 0.35 h)
1
14
24
38
52
0
24
48

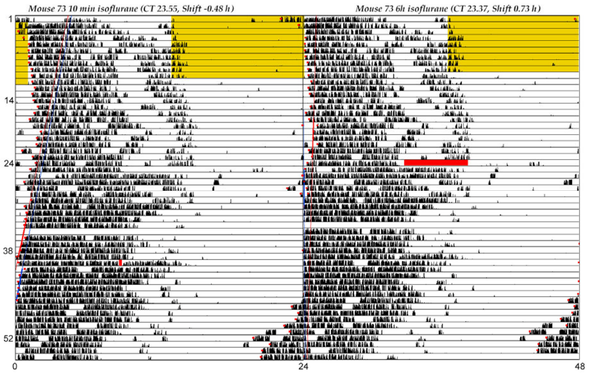
Mouse 73 10 min isoflurane (CT 23.55, Shift -0.48 h)
Mouse 73 6h isoflurane (CT 23.37, Shift 0.73 h)
1
14
24
38
52
0
24
48

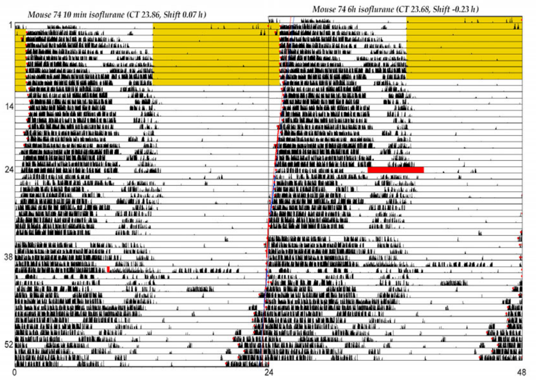
Mouse 74 10 min isoflurane (CT 23.86, Shift 0.07 h)
Mouse 74 6h isoflurane (CT 23.68, Shift -0.23 h)
1
14
24
38
52
0
24
48

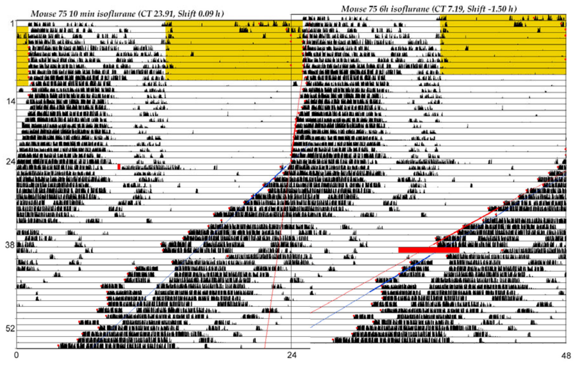
Mouse 75 10 min isoflurane (CT 23.91, Shift 0.09 h)
Mouse 75 6h isoflurane (CT 7.19, Shift -1.50 h)
1
14
24
38
52
0
24
48

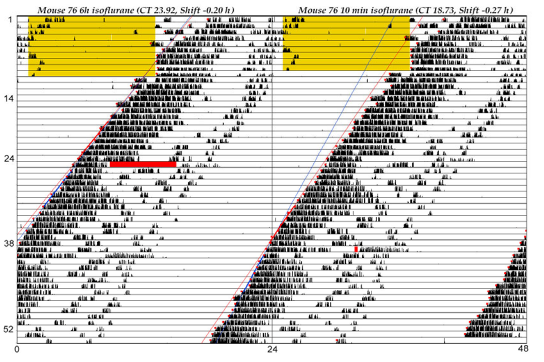
Mouse 76 6h isoflurane (CT 23.92, Shift -0.20 h)
Mouse 76 10 min isoflurane (CT 18.73, Shift -0.27 h)
1
14
24
38
52
0
24
48

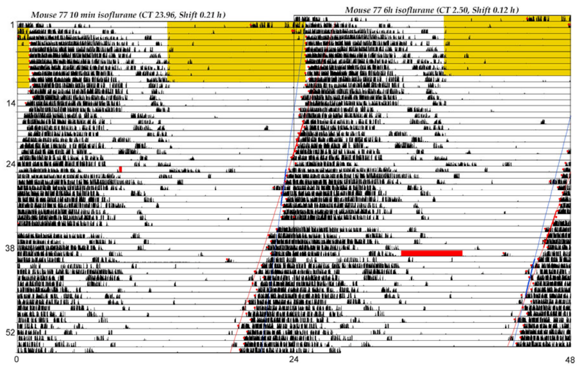
Mouse 77 10 min isoflurane (CT 23.96, Shift 0.21 h)
Mouse 77 6h isoflurane (CT 2.50, Shift 0.12 h)
1
14
24
38
52
0
24
48

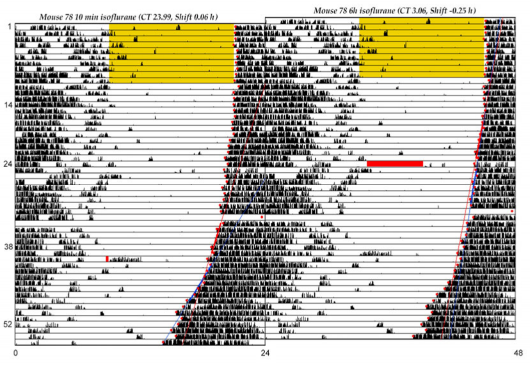
Mouse 78 10 min isoflurane (CT 23.99, Shift 0.06 h)
Mouse 78 6h isoflurane (CT 3.06, Shift -0.25 h)
1
14
24
38
52
0
24
48

Mouse 79 10 min isoflurane (No shift calculated)

Mouse 79 6h isoflurane (CT 7.09, Shift 1.1 h)

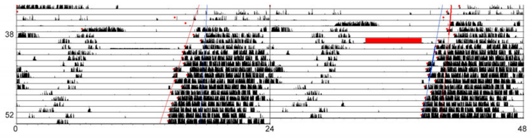

Mouse 80 10 min isoflurane (No phase shift calculated)

Mouse 80 6h isoflurane (CT 13.71, Shift -0.74 h)

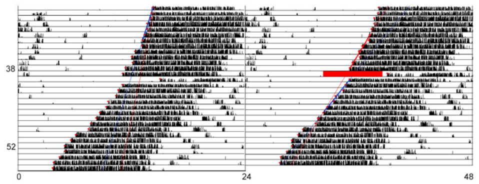

Supplement: Supplementary file 1 [file clockssleep-03-00006-s001.pdf]
